# Using deep learning to predict the outcome of live birth from more than 10,000 embryo data

**DOI:** 10.1186/s12884-021-04373-5

**Published:** 2022-01-16

**Authors:** Bo Huang, Shunyuan Zheng, Bingxin Ma, Yongle Yang, Shengping Zhang, Lei Jin

**Affiliations:** 1 Reproductive Medicine Center, Tongji Hospital, Tongji Medicine College, Huazhong University of Science and Technology, Wuhan, People’s Republic of China; 2 School of Computer Science and Technology, Harbin Institute of Technology, Weihai, 264209 China

**Keywords:** Time-lapse microscopy, Embryo development, Embryo quality, Pregnancy

## Abstract

**Background:**

Recently, the combination of deep learning and time-lapse imaging provides an objective, standard and scientific solution for embryo selection. However, the reported studies were based on blastocyst formation or clinical pregnancy as the end point. To the best of our knowledge, there is no predictive model that uses the outcome of live birth as the predictive end point. Can a deep learning model predict the probability of live birth from time-lapse system?

**Methods:**

This study retrospectively analyzed the time-lapse data and live birth outcomes of embryos samples from January 2018 to November 2019. We used the SGD optimizer with an initial learning rate of 0.025 and cosine learning rate reduction strategy. The network is randomly initialized and trained for 200 epochs from scratch. The model is quantitively evaluated over a hold-out test and a 5-fold cross-validation by the average area under the curve (AUC) of the receiver operating characteristic (ROC) curve.

**Results:**

The deep learning model was able to predict live birth outcomes from time-lapse images with an AUC of 0.968 in 5-fold stratified cross-validation.

**Conclusions:**

This research reported a deep learning model that predicts the live birth outcome of a single blastocyst transfer. This efficient model for predicting the outcome of live births can automatically analyze the time-lapse images of the patient’s embryos without the need for manual embryo annotation and evaluation, and then give a live birth prediction score for each embryo, and sort the embryos by the predicted value.

**Supplementary Information:**

The online version contains supplementary material available at 10.1186/s12884-021-04373-5.

## Introduction

Since Louis Brown was born, the first test tube baby [1], more than seven million babies have been born around the world attribute to assisted reproduction technology (ART) [2]. In the early stage of IVF technology development, multiple embryo transfer was the main transfer method. However, multiple pregnancy was often accompanied by premature delivery, more expenditure and higher risk of complications [3–6]. Therefore, with the development of assisted reproductive technology, single embryo transfer has gradually become the first choice of IVF. However, single embryo transfer still faces an urgent problem: how to choose the best embryo to transfer to maintain the ideal success rate [7]. The trend of choosing single embryo transfer is closely related to the improvement and progress of embryo selection technology. Therefore, embryo identification and selection technology are particularly important and significant. In order to solve this problem, scholars have developed several methods for identifying and selecting the best embryos for transfer, such as: blastocyst culture, time-lapse photography imaging system and pre-transfer genetic testing [8–10].

Embryologists evaluated and observed the embryos used optical microscope, which was taken out from the conventional incubator at a specific time point during the first 5 days of life before the time-lapse imaging system was applied to the clinic [11]. Because of this disadvantage, many events in the embryonic development process have been missed [12]. And the emergence of time-lapse photography technology had just made up for this shortcoming.

Embryologists use the time-lapse photography system to observe and evaluate the embryo that in a stable environment, rather than exposed in a variable condition (such as changing gas composition, unstable humidity, insecure temperature and movement conditions), and can obtain a lot of information between embryo development, time and embryo potential [13, 14].

Scholars have introduced the mathematical technology of artificial intelligence into ART, in order to acquire more information from the pictures obtained by the TL system, which may trigger a revolution. AI is a term that can be divided into many areas, such as: artificial neural network (ANN), fuzzy logic, genetic algorithm (GA), machine learning and deep learning [15, 16].

The emergence of time-lapse incubation makes it possible to record the complete cycle of an embryo from a blastomere to a blastocyst, when all morphokinetic features centralized [17]. Meanwhile, owing to its abundant time-lapse data, time-lapse incubation emerges up many new research ideas combined with deep learning technology which is known as a data driven method. Deep learning can uncover numerous subtle features which may not be paid attention to manually but do help the corresponding classification or prediction. When fed with enough well labeled data, deep learning model have the ability to find an optimal representation of the given dataset by continuously conducting back-propagation. Thus, we can explore the general pattern which lead to a specific mapping from data to our desired tasks.

The deep learning literature that has been reported on embryo selection is a design study with blastocyst formation or clinical pregnancy as the end point. To the best of our knowledge, there is no research on deep learning models designed with the end of live birth outcome. In this study, we want to analyze the data of single-center, large sample of single blastocyst transfer to obtain an efficient predictive model.

## Materials and methods

### Patients

This was a noninterventional, retrospective, single-center cohort study of patients undergoing routine practice. In order to reflect the broad range of patients typically encountered in clinical practice, no inclusion/exclusion criteria were applied on baseline characteristics. The time-lapse embryo data used in our work are collected from Reproductive Medicine Center of Tongji Hospital, Huazhong University of Science and Technology, Wuhan, China. The whole dataset contains 33,738 embryo samples captured by Embryoscope Plus time-lapse microscope system. The fertilization time of these embryos were from January 2018 to November 2019, and we continuously pay return visits until January 2021 to confirm whether these IVF treatments lead to live birth outcomes. All patients signed written informed consent and underwent the routine clinical treatment performed in our center. No additional intervention was performed.

#### Ethical approval

The study conformed to the Declaration of Helsinki for Medical Research involving Human Subjects. It was approved by the Ethical Committee of Reproductive Medicine Center, Tongji Hospital, Tongji Medicine College, Huazhong University of Science and Technology.

#### Dataset

**Fig. 1:**
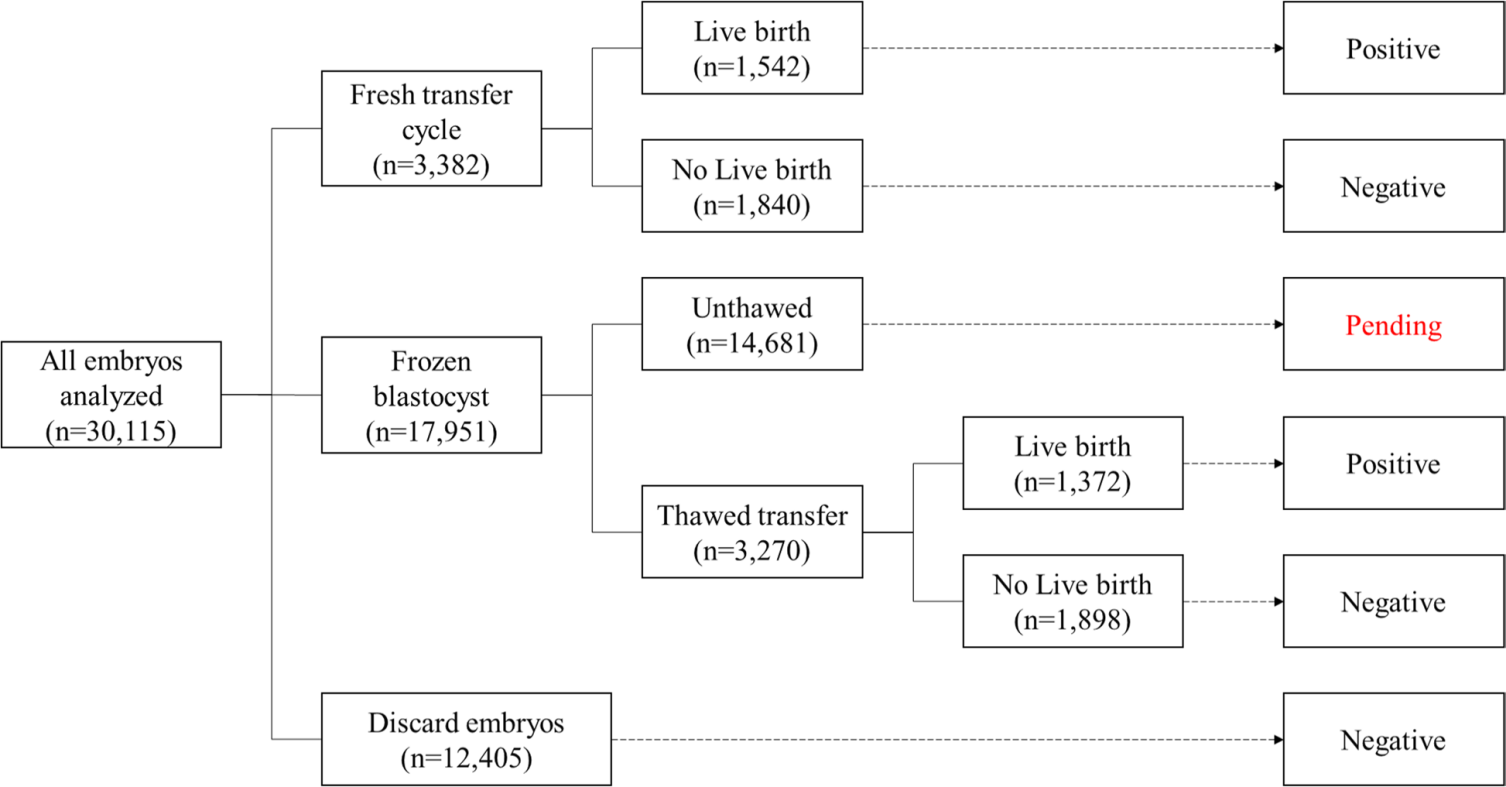
The outcomes of the embryos being studied

The classification of the outcome of each embryo was shown in Table 1. And the final indicator was live birth. The whole dataset contained 33,738 embryos with labels of positive, negative, and pending, as shown in Fig. 1. The pending embryos referred to the unthawed embryos which could be exploited in our future work, but were excluded in the experiments of this paper. Meanwhile, only the single blastocyst transfer embryos were collected, including fresh cycle and frozen-thaw cycle. Thus, the engaged dataset in this paper contained 15,434 embryos with positive and negative labels.

**Table 1:** Classification of the outcome of each embryo involved

Classification	Outcome
Positive	Live birth after a complete pregnancy cycle
Negative	Fail to live birth or embryo discarded because of a failed or abnormal fertilization, grossly abnormal morphology or aneuploidy from preimplantation genetic testing
Pending	Embryo in storage and not yet used

#### Embryo culture and frozen-embryo transfer (FET)

The methods used for sperm preparation, for IVF and embryo culture, have been described previously [18]. Briefly, semen was collected in sterile containers by masturbation after 3–5 d of sexual abstinence and then maintained at 37 °C for 30 min. After liquefaction, samples were analyzed for sperm concentration, motility and morphology according to the World Health Organization criteria. The oocytes were incubated in G-IVF medium (Vitrolife) and fertilized 3 to 4 h after retrieval. Normal fertilization was defined as zygotes with two pronuclei (2PN) and fertilized oocytes were continuously cultured in G1 medium for 2 more days. Then, the embryos were transferred to G2 medium and continued to be cultured for 3 more days. The additional good-quality blastocysts were cryopreserved for subsequent frozen-embryo transfer (FET) cycles. For the FET cycles, oral estradiol (Progynova, Bayer) was provided, 2 mg/d from cycle day 1–4, 4 mg/d from day 5–8 and 6 mg/d from day 9–12. Transvaginal ultrasound scanning was performed to assess the endometrial thickness and ovulation from day 13; the estradiol dosage was adjusted based on the endometrial thickness. Administration of 40 mg progesterone intramuscularly was given when the endometrium reached a thickness of 8 mm or maximum. Administration of 60–80 mg of progesterone was provided for the following 5 days. Blastocysts transfer was performed on day 6, after 5 days of progesterone administration.

Serum hCG was measured to diagnosis a pregnancy 2 weeks after embryo transfer and then was tested serially to monitor rising titers. A clinical pregnancy was defined as the presence of a gestational sac with fetal heart activity observed on ultrasound examination 5 weeks after oocyte retrieval [19]. The live birth outcome data were obtained by telephone interview of the parents after delivery.

#### Deep learning model

In this work, we designed an end-to-end deep learning model to predict live birth probability. We label our embryo samples by 0 and 1 according to real live birth outcomes, where 1 represents live birth whereas 0 represents not. The designed supervised network regresses the discrete prediction value between 0 and 1 under the guidance of ground truth labels.

The network structure consists of seven convolution modules and two fully connected layers. The first module contains three convolution blocks which represents a combination of a convolution layer, a batch normalization layer and a following ReLU (Rectified Linear Unit) as an activation function. As is widely known that the residual block proposed in ResNet [20] is demonstrated effective in numerous classification tasks, the subsequent six convolution modules who share the same architecture are composed of three basic residual blocks and a convolution block. Feature maps are down sampled at the last convolution block of each module. The whole network in this work can be described a ResNet like network, as shown in Table 2. but the number of modules differs from that in benchmark structure. Also, the complexity of our model is much higher than the benchmark model, specifically reflected on the number of convolution kernels.

**Table 2:** Network structure of the proposed method. The basic block is engaged from ResNet18 [20]

Layer	Filter Size	Output Size
Conv1_x	7 × 7, 64 3 × 3, 64 3 × 3, 128, *stride* 2	224 × 224 224 × 224 112 × 112
Conv2_x	$$\left[\begin{array}{c}3\times 3,128\\ {}3\times 3,128\end{array}\right]\times 3$$ 3 × 3, 256, *stride* 2	112 × 112 56 × 56
Conv3_x	$$\left[\begin{array}{c}3\times 3,256\\ {}3\times 3,256\end{array}\right]\times 3$$ 3 × 3, 512, *stride* 2	56 × 56 28 × 28
Conv4_x	$$\left[\begin{array}{c}3\times 3,512\\ {}3\times 3,512\end{array}\right]\times 3$$ 3 × 3, 1024, *stride* 2	28 × 28 14 × 14
Conv5_x	$$\left[\begin{array}{c}3\times 3,1024\\ {}3\times 3,1024\end{array}\right]\times 3$$ 3 × 3, 2048, *stride* 2	14 × 14 7 × 7
Conv6_x	$$\left[\begin{array}{c}3\times 3,2048\\ {}3\times 3,2048\end{array}\right]\times 2$$ 3 × 3, 2048	7 × 7 5 × 5
Conv7_x	$$\left[\begin{array}{c}3\times 3,2048\\ {}3\times 3,2048\end{array}\right]\times 2$$ 3 × 3, 2048	5 × 5 3 × 3
Fc1	Max pool 3 × 3 2048-d fc	1 × 1

We utilize BCE-Loss (binary cross entropy loss) as a loss function to guide the backpropagation during training term when the model constantly optimizes itself. Since the loss function calculate the distance between output predictions and target labels, our purpose is to minimize the loss value.

#### Training strategies

Aimed at the extremely imbalance of the positive and negative samples, we implement the following measures during the training term. In the cross-validation experiment, we perform data augmentation after splitting the dataset according to Table 3. The specific method is as follows: Firstly, we conduct abundant data augmentation measures, including affine transformations and randomly coarse dropout. Affine transformations refer to flip, translation, rotation, scaling, each operation occurs randomly at a probability of 50 %. Coarse dropout means randomly drop some local pixels, the selected local pixels are painted in solid black, we set the probability ranging from 2 to 5%. Secondly, we over sample the positive samples at a certain multiple, which equals to the ratio of positive and negative samples, i.e., sixteen in our experiments. The original images captured by time-lapse incubation are 800^2^ pixels, which should be further resized to 224^2^ for network training after data augmentation.

**Table 3:** Result of the 5-fold cross-validation analysis

	*Fold 1* *(n = 3812)*	*Fold 2* *(n = 3812)*	*Fold 3* *(n = 3811)*	*Fold 4* *(n = 3811)*	*Fold 5* *(n = 3811)*	*AUC*
*1*	*Test*	*Train*	*Train*	*Train*	*Train*	*0.970*
*2*	*Train*	*Test*	*Train*	*Train*	*Train*	*0.964*
*3*	*Train*	*Train*	*Test*	*Train*	*Train*	*0.968*
*4*	*Train*	*Train*	*Train*	*Test*	*Train*	*0.976*
*5*	*Train*	*Train*	*Train*	*Train*	*Test*	*0.960*
***Average***						*0.968*

Average AUC, The mean area under the curve across 5 cross-validation steps

We used the SGD optimizer with an initial learning rate of 0.025 and cosine learning rate reduction strategy. The network is randomly initialized and trained for 200 epochs from scratch.

#### Performance testing

The model is quantitively evaluated over a 5-fold cross-validation by the average area under the curve (AUC) of the receiver operating characteristic (ROC) curve.

ROC curve connects all points described by true positive rate and false positive rate under all possible thresholds, which is a boundary value between positive and negative samples. Considering that true positive rate and false positive rate are in a trade-off relationship corresponding to thresholds, we can quantify the discriminating power by calculating the area under the curve, this is so-called AUC. A binary classifier who has incomparable discriminating power can possess an AUC value of 1, whereas the weakest who almost emerge the judgement randomly possess an AUC value of 0.5, and a higher AUC value implies a better performance. AUC is more reasonable than accuracy especially in classification tasks with imbalance data.

In order to comprehensively evaluate the performance of our model, we perform a hold-out test and a 5-fold cross-validation simultaneously [21]. In the hold-out test or so-called train-val-test approach, we randomly split the dataset in a ratio of 5:1:1 for training set, validation set, and test set, respectively. In the latter evaluation method, we randomly divide our data into five parts with equal size, where the proportion of positive and negative samples in each separate is same. Then, five models should be trained. In each case, a specific subset is selected for validation while the remaining four subsets serve as a training set. Finally, we can figure out the mean AUC of the five folds to evaluate the performance on the whole dataset. Compared with hold-out test, cross-validation can eliminate the possible overestimating or underestimating caused by undesired sample division.

## Results

From January 2018 to November 2019, a total of 5913 cycles used the time-lapse culture system. Among them, some patients have not been transferred in fresh cycle, and their embryos have not yet been thawed. In the end, 3382 fresh cycles and 3270 frozen-thaw transfer (FET) cycles were included in the study and 33,738 embryos samples were analyzed. Basic information of the patients included in this study was shown in Table 4.

**Table 4:** Basic information of the patients included in this study

Age (y)	30.4 ± 3.9
Duration of infertility (y)	3.3 ± 2.3
Duration of stimulation (d)	10.4 ± 1.8
Basal FSH (IU/L)	7.4 ± 1.9
No. of oocytes retrieved	12.9 ± 4.2
No. of mature oocytes	11.0 ± 3.8
No. of embryo cultured	7.9 ± 3.5

### Roc

**Fig. 2:**
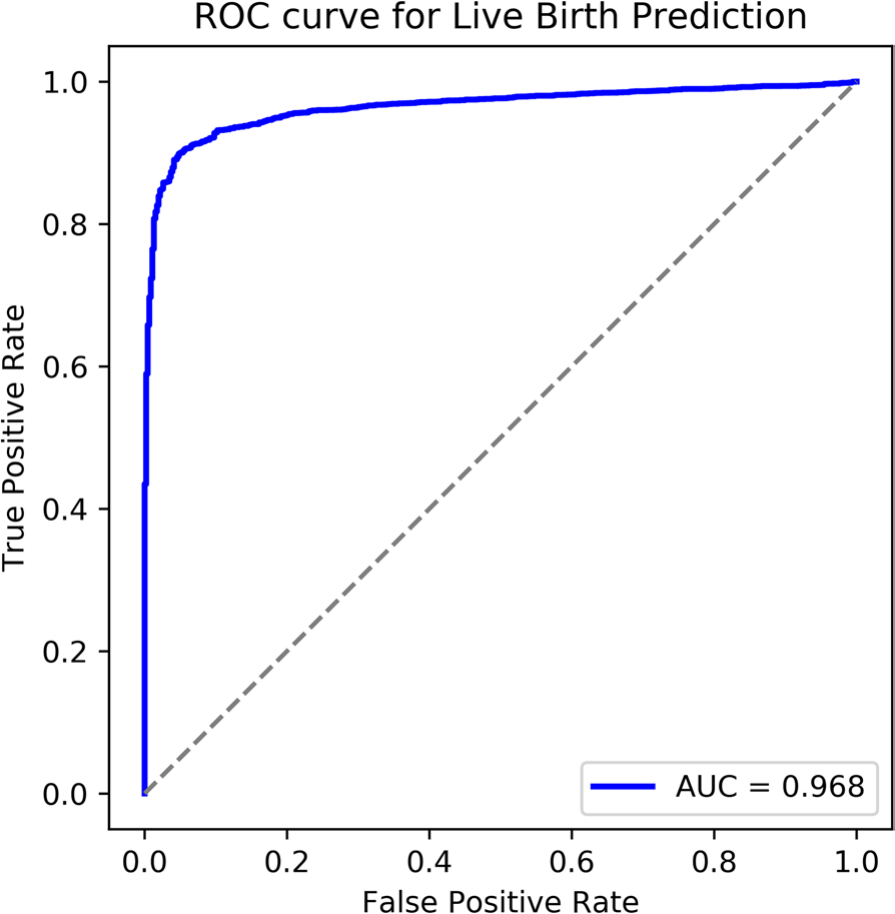
ROC curve for prediction of live birth. ROC, Receiver operating characteristic. AUC, area under the curve

Analysis of the ROC was shown in Fig. 2. The resulting AUC of this research to predict live birth on the testing dataset was 0.968.

### 5-fold cross-validation

Table 3 showed the results of 5-fold cross-validation. The average value of AUC was 0.968. The AUC was reproducible in individual train-validation runs.

### Hold-out test

The AUC value of the conducted hold-out test was 0.957, which was evaluated on the test set. The result was comparable with the 5-fold cross-validation.

## Discussion

This study is a preliminary study of deep learning with live birth data as the end point during the IVF cycle. Our results show that Timelapse images can be combined with deep learning technology for clinical applications.

Morales et al. [22], Xu et al. [23] and Santos Filho et al. [24] used static images to assess embryo quality or select the best embryos to be transferred in the absence of early embryo development data. These methods lack support of more comprehensive data.

Dirvanauskas et al. [25] used convolutional neural network (CNN) to predict the developmental stage of the embryo analyze by analyze embryo images obtained from the time-lapse photography system, with a success rate of 97.62%. However, this method does not have the ability to predict pregnancy. Khosravi et al. developed a new framework (STORK) based on the inception of Google’s model to predict the quality of embryos with an AUC as high as 0.98. The study has a large sample size, complex model, and high accuracy, but it cannot be used to predict live births [26]. It is demonstrated that our model has a better performance when compared with existing benchmark model, but it still deserves to be optimized since it’s high complexity. Such a complex network requires considerable computing resources, so it depends highly on hardware device.

There is also a latest report that creates the predictive model of blastocyst transfer [27]. The author analyzed the data of more than 10,000 embryos and obtained a predictive model with an AUC of 0.93. However, the predictive endpoint of this study is the clinical pregnancy, which is the most prominent difference from our study. In this study, we hope to get the best predictive effect, so we chose to predict the blastocyst transfer based on the final live birth outcome.

Obviously, there is no single method that can solve all the problems in the field of assisted reproduction, and different methods have their own key research directions. The model we developed was very complex and has a high accuracy rate. That includes a large sample size, and the sample database covers patients and clinical programs with various conditions. The results are repeatable and have high clinical guidance significance. However, we have to admit that our data come from the embryo images obtained by the time-lapse photography system after fertilization, ranging from 105 h to 125 h, instead of video data which lacks early embryo development data. If we generalize this model into the task of prediction from 3-day embryos, more refined works need to be done. As we all known, more spatiotemporal features can be captured if we use the entire video as an input. But we find the predictive power will not progress obviously if we use the whole video as input rather than the blastocyst frames, considering the parameters of a model are greatly restricted due to the capacity of machines when faced with video data.

There is no clear evidence that AI applied to IVF can increase the cumulative success rate [28, 29]. Whether a patient can finally give birth to a healthy baby is not only related to the embryo itself, but also to the patient’s own health, age, reproductive history, clinical plan and many other factors. Our deep learning model does not include these variables in the database, which is also the direction we need to work hard in the future. It is worth noting that the live birth rate in this study showed a high level (45.6%). As we all know, age and ovarian reserve are very important factors that determine the clinical pregnancy rate and live birth rate of IVF [30]. This higher live birth rate may be related to the younger population in this study (average age is 30.4 years) and better ovarian reserve (average number of oocytes retrieved is 12.9).

In 2019, an important paper was reported in AI-assisted embryo selection, the author retrospectively analyzed time-lapse videos and clinical outcomes of 10,638 embryos from eight different IVF clinics [31]. The deep learning model they reported was able to predict fetal heart pregnancy from time-lapse videos with an AUC of 0.93. We think our research is different. This article is a single-center research. The advantage of this lies in the data analysis of large samples in a single center, which avoids the influence of different embryo operation procedures and different embryo culture systems. On the other hand, we directly used the live birth outcome as the deep learning model label. The false positive data of aborted embryos can be excluded.

There is another flaw in this study, that is, the samples are all from blastocyst transfer, and there is no model design for cleavage embryo transfer. In fact, we have tried deep learning for the evolution of the cleavage stage, but the effect is not satisfactory. This may be one of the reasons why there is no model reported for predicting the outcome of the cleavage stage embryo [27, 31, 32].

In conclusion, this model has good predictive value for embryos selection by deep learning. It can help embryologists choose the best embryos for transfer, freezing and thaw, and can shorten the time for patients from embryo transfer to becoming a parent.

## Supplementary Information

**Additional file 1.** A brief explanatory diagram of this research.

**Additional file 2.** A brief description video of this research.

## Data Availability

The datasets used and/or analysed during the current study are available from the corresponding author on reasonable request.
