# Supplementary figures and images for: Using deep learning to predict the outcome of live birth from more than 10,000 embryo data
Source: BMC Pregnancy Childbirth. 2022 Jan 16;22:36. doi: 10.1186/s12884-021-04373-5 (PMC8761300; doi:10.1186/s12884-021-04373-5)

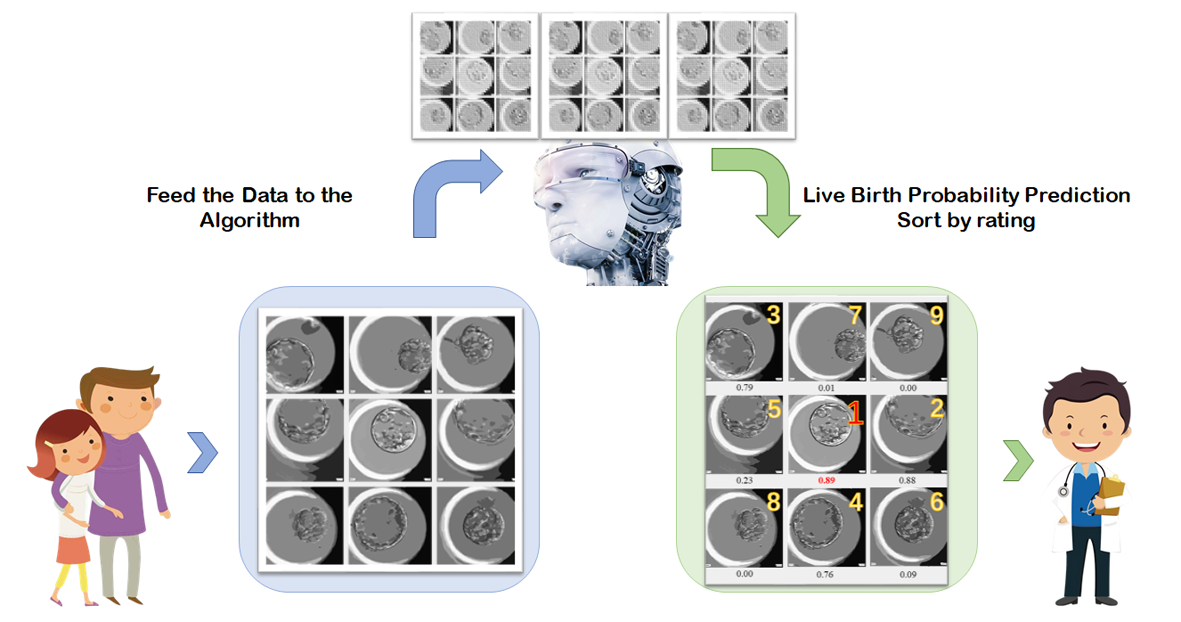

Supplement: Supplementary file 1 — Additional file 1. A brief explanatory diagram of this research. [file 12884_2021_4373_MOESM1_ESM.tif]
